# The efficacy and safety of 0.01% atropine alone or combined with orthokeratology for children with myopia: A meta-analysis

**DOI:** 10.1371/journal.pone.0282286

**Published:** 2023-07-26

**Authors:** Zengying Wang, Pengfei Wang, Bohua Jiang, Yifei Meng, Sufang Qie, Zhipeng Yan

**Affiliations:** The Third Hospital of Hebei Medical University, Shijiazhuang, Hebei Province, China; Xiamen University, CHINA

## Abstract

**Objective:**

To evaluate the efficacy and safety of 0.01% atropine alone and in combination with orthokeratology for myopia control using a meta-analysis.

**Methods:**

PubMed, Cochrane Library, and EMBASE were searched. We included eligible randomized controlled trials (RCTs), non-RCTs, and retrospective cohort studies, published up to August 1, 2022. We calculated the weighted mean difference (WMD) and 95% confidence interval (CI) for all outcomes and plotted them in forest plots.

**Results:**

Fourteen studies were included; 4 and 11 in the 0.01% atropine monotherapy and atropine-orthokeratology (AOK) groups, respectively. Compared with orthokeratology (OK) alone, 0.01% atropine alone had similar effects on slowing the axial elongation (WMD: −0.00 mm; 95% CI: −0.05–0.04, p<0.31), while AOK significantly lowered axial growth. Moreover, the baseline myopic degree and duration of treatment were influential for the change in axial elongation (WMD: −0.12 mm; 95% CI: −0.17–−0.07, p = 0.00001 and WMD: −0.11 mm; 95% CI: −0.15–−0.108, p<0.00001, respectively). Additionally, the AOK may reduce the change rate of the spherical equivalent refraction and the accommodation amplitude (WMD: −0.13 D; 95% CI: 0.07–0.19, p<0.001 and WMD: −1.08 mm; 95% CI: −1.73–−0.43, p<0.0001, respectively), and cause a slight increase in the diameter of the pupil (WMD: 0.56 mm; 95% CI: 0.43–0.70, p = 0.007). No significant differences in the uncorrected distant visual acuity, best corrected visual acuity, intraocular pressure, tear film break-up time, lipid layer thickness, and corneal endothelial cell density were found between the OK and AOK groups.

**Conclusion:**

In slowing the axial elongation, 0.01% atropine alone and OK alone have similar effects, while AOK is more effective than OK alone in slowing down the axial elongation. Furthermore, the baseline degree of myopia and treatment duration may affect changes in axial elongation.

## Introduction

Myopia is among the most common eye diseases globally. The prevalence in the adult population is 10%–30% in many countries. In some parts of East and Southeast Asia, the prevalence in young adults is 80%–90% [1]. The development of severe myopia leads to excessive axial growth, and severe and irreversible vision loss including retinal detachment [2], macular dystrophy, and glaucoma [3]. Therefore, it is particularly important to take effective measures to slow the axial elongation to control the progression of myopia.

Among the current myopic interventions, atropine eye drops and orthokeratology (ortho-K) are considered effective in inhibiting the progression of myopia [4]. At present, the mechanism by which atropine controls myopia progression is not clear. Studies have suggested that atropine can slow myopia progression through a non-regulatory mechanism. It may act on M1 and M4 subtypes of the muscarinic receptors of the retina and sclera and slow the growth of the axial length, and may also promote the release of dopamine and other neurotransmitters, thus affecting the signal transduction of the retina and slow the growth of the axial length [5]. The atropine for the treatment of childhood myopia studies 1 and 2 have suggested that atropine has a dose-dependent clinical efficacy in controlling myopia progression and that low-concentration atropine was the most appropriate dosage, with fewer side effects of pupil dilation, photophobia, and cycloplegia [6, 7]. At present, 0.01% atropine is most commonly used for myopia control in East Asia, especially in Taiwan and Singapore [8]. However, atropine treatment must be combined with corrective spectacles, contact lenses, and orthokeratology (OK) lenses to provide refractive correction.

Orthokeratology (OK) is a special type of rigid contact lens with a reverse geometric design. These are worn at night to change the corneal curvature in patients, thus, reversibly reducing the degree of myopia and improving daytime vision [9]. The mechanism of the OK lens involves corneal epithelial remodeling (thinning of the central cornea and thickening of the peripheral cornea), which causes myopia defocusing in the peripheral retina, thus, controlling the progression of myopia [10]. Most studies have confirmed that OK can reduce axial elongation by 32%-63% over two years [11].

Some studies have compared atropine alone and OK alone for juvenile myopia, but the results remain controversial [12–14]. Due to the different mechanisms of atropine and OK in controlling myopia progression, the combination of atropine and OK may produce an additive effect. Several studies have confirmed that the combination of 0.01% atropine and OK is more effective than OK alone in slowing axial elongation in children with myopia [15–25]. In this meta-analysis, we aimed to further evaluate the efficacy and safety of 0.01% atropine alone and in combination with orthokeratology for myopia control.

## Methods

### Search strategy

The databases, PubMed, Cochrane Library, and EMBASE, were searched. Potentially eligible randomized controlled trials (RCTs), non-randomized controlled trials (non-RCTs), and retrospective cohort studies (REs) published from the establishment of the database to August 1, 2022, were considered. The search terms, using Medical Subject Headings and free words, were as follows: orthokeratology procedures, orthokeratology, ortho-k, OK lens, orthokeratology lens, atropine, atropine sulfate, myopia, near sight, refractive errors, and nearsightedness. The primary treatment efficacy was measured via the mean change in axial length (AL) and spherical equivalent refraction (SER) after therapy. The primary measures of treatment safety included the mean change in uncorrected distant visual acuity (UCVA), best corrected visual acuity (BCVA), pupil diameter (PD), accommodation amplitude (AA), intraocular pressure (IOP), tear film break-up time (TBUT), lipid layer thickness (LLT), and corneal endothelial cell density (CECD). Furthermore, the reference lists of published reviews were carefully screened to identify applicable studies.

### Eligibility criteria

We selected all studies according to the criteria as follows. (1) Study population: children, aged under 18 years, with myopia, equivalent spherical refraction of less than -6.0D, and binocular anisometropia of less than -1.5D at baseline. (2) Intervention measures: the experimental group was treated with 0.01% atropine combined with OK or 0.01% atropine alone, whereas the control group received OK only. (3) Study Design: RCTs, non-RCTs, and REs. (4) The study described at least one of the following outcomes: a mean change in AL, SER, UCDA, BCDA, PD, AA, IOP, TBUT, LLT, or CECD after treatment, and which was statistically significant. (5) Treatment duration: at least 6 months. Studies that did not meet the inclusion criteria, or had incorrect or incomplete data, conference reviews, case reports, and duplicate publications were excluded.

### Data extraction and quality assessment

Two reviewers (WZY and YZP) independently performed data extraction and quality assessment. All extracted data were recorded in a Microsoft Excel spreadsheet including first author, year of publication, study design, intervention, follow-up time, baseline participant characteristics (age, sample size, AL, and SER), and measurement methods. The RCT quality was evaluated according to the Cochrane Collaboration’s tool for assessing the risk of bias [26], which included seven domains as follows: random sequence generation, allocation concealment, blinding of participants and personnel, blinding of outcome assessment, incomplete outcome data, selective reporting, and other biases. In each domain, the reviewers assessed each domain as “low risk” of bias, “high risk” of bias, or “clear risk” of bias, according to the given criteria. If insufficient details are reported in the study, an “unclear risk” of bias was considered. We used the Newcastle-Ottawa Scale to assess the quality of the observational studies [27]. A score of seven or more on the Newcastle-Ottawa Scale was considered high quality, four to six as moderate quality, and less than three as low quality. The quality of each included non-RCT was assessed based on the methodological index for non-randomized studies (MINORS) [28]. Disagreements were resolved by discussion with third-party experts or by focused discussions that involved at least three reviewers.

### Statistical analysis

Statistical analyses were performed using Review Manager (version 5.3). The weighted mean difference (WMD), and 95% confidence interval (CI), were calculated for all the outcomes. Heterogeneity was assessed using I2 statistics. If I2 was ≥50%, the random-effects model was used for meta-analysis. Otherwise, the fixed-effects model was chosen. Sensitivity analysis was performed by sequentially eliminating one study. Publication bias was detected using a funnel plot test.

## Results

### Literature search and characteristics of the included studies

**Fig 1:**
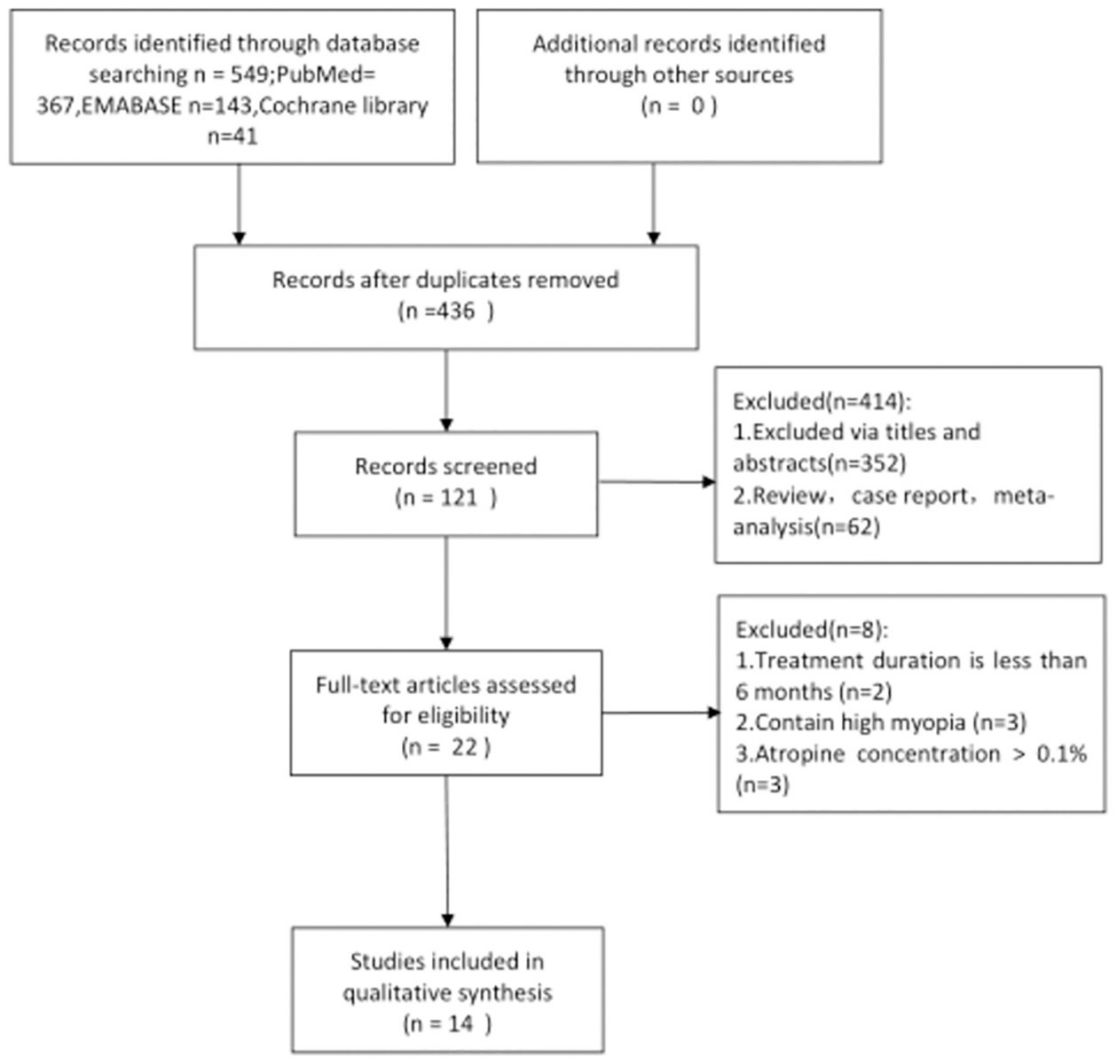
A flow diagram of the included studies eligible for meta-analysis.

A total of 549 studies were obtained from the searched databases based on the specified search strategy. After eliminating 111 duplicates, we reviewed the abstracts of 436 studies, of which 414 were irrelevant. The final 22 studies were reviewed in full, and 8 were excluded because they did not meet our inclusion criteria. Finally, 14 studies were included in this meta-analysis, including 8 RCTs, 3 non-RCTs, and 3 REs. The screening process for eligible studies is shown in a flow diagram in Fig 1.

Table 1 presents the characteristics of the 14 eligible studies. Four studies were categorized into the 0.01% atropine monotherapy group [12–14, 25], and 11 were classified into the AOK group [15–25]. The study by Zhao (2021) [25] was included in both the 0.01% atropine monotherapy and AOK groups. Two studies (Kinoshita, 2018 [18] and Kinoshita, 2020 [17]) were conducted in Japan, and the remainder were conducted in China. A total of 1230 children with myopia were included in this study. Of the 1230 children, 395 were in the 0.01% atropine monotherapy group and 865 were in the AOK group. In the 0.01% atropine monotherapy group, the children were prescribed single-vision spectacle lenses that were worn constantly, in addition to administering one drop of 0.01% atropine in each eye before bedtime every night. In 11 studies, self-prepared low-concentration atropine was used, while in three studies, low-concentration, preservative-free atropine eye drops were used.

**Table 1:** Baseline characteristics of all included studies.

First author	Country	Study design	Sample size(E/C)	Age(years)	Group	Follow-up(month)	SER,D(E/C)	Axial Length, mm(E/C)	Axial Length Measurements
Ji N2022	China	RE	46/49	9.96±1.75/10.14±1.84	SA/OK	12	H:-3.78±1.21/-4.01±1.13	H: 24.71±0.89/24.80±0.81	IOL master
							L: -2.18±1.08/-1.94±1.33	L:24.01±0.84/24.08±1.32	
Jiang J2018	China	Non-RCT	80/80	11.50±2.30/12.10±2.10	SA/OK	18	-2.85±0.54/-2.75±0.52	23.90±1.30/23.93±1.07	-
Ren QL2017	China	RCT	50/50	11.96±2.58	SA/OK	12	-3.16±0.28/-3.08±0.11	24.32±1.16/24.28±1.32	-
Zhao Q2021	China	RCT	20/20	9.65±1.53/11.00±1.17	SA/OK	6,12	-1.98±0.45/-2.75±0.46	24.17±0.68/24.42±0.48	LS-900
Zhao Q2022	China	RCT	20/21	10.9±1.29/11.00±1.17	AOK/OK	6,13	-2.85±0.45/-2.75±0.46	24.56±0.39/24.42±0.48	LS-900
Tan Q2020	China	RCT	29/30	9.20±1.00/9.10±1.20	AOK/OK	6,12	-2.65±0.92/-2.84±0.96	24.43±0.62/24.43±0.81	IOL master
Tan Q2022	China	RCT	34/35	9.20±1.00/9.10±1.20	AOK/OK	6,12,24	-2.76±0.88/-2.83±1.01	24.56±0.71/24.50±0.92	IOL master
Chen Z2019	China	RE	28/29	7.50±1.20/8.30±1.50	AOK/OK	12	-2.70±1.10/-2.50±0.94	24.80±0.87/24.53±0.61	IOL master
Chen Z2021	China	RE	37/36	8.90±1.40/8.80±1.21	AOK/OK	12,24	-2.85±1.08/-2.38±1.10	24.49±0.96/24.26±0.90	IOL master
Kinoshita N2018	Japan	RCT	20/20	10.87±1.38/10.40±1.86	AOK/OK	12	− 2.81±1.43/− 2.95±1.43	24.73±0.58/24.95±0.92	IOL master
Kinoshita N2020	Japan	RCT	38/35	10.33±1.59/10.37±1.65	AOK/OK	24	-2.60±1.29/-2.72±1.31	24.69±0.58/24.86±0.81	IOL master
Vincent J2020	China	RCT	25/38	8.90±1.20/9.10±1.10	AOK/OK	6	-2.38±0.81/-2.58±0.91	24.38±0.62/24.44±0.84	IOL master
Tang WT2020	China	RCT	L: 20/22 H: 43/41	11.05±2.13	AOK/OK	12	L:-2.56±1.15/-2.59±1.12	L:23.72±0.31/23.70±0.29	IOL master
							H:-4.90±1.16/-4.92±1.21	H:24.69±0.34/24.71±0.37	
Niu YL2019	China	Non-RCT	80/72	13.73±2.52/13.61±2.41	AOK/OK	12	-1.40±0.25/-1.38±0.23	23.58±3.39/23.61±3.42	IOL master
Luo Y2021	China	Non-RCT	60/60	10.02±2.35/10.77±1.91	AOK/OK	12	-3.26±1.35/-3.18±1.32	25.12±0.81/25.01±0.79	IOL master

### Bias risk assessment

**Fig 2:**
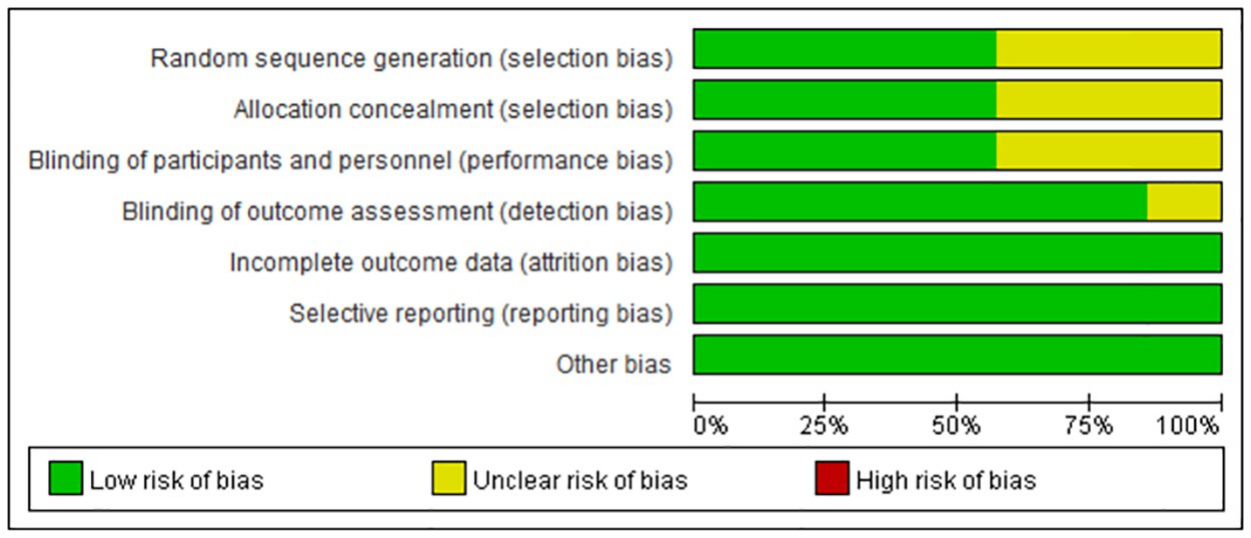
Risk of bias graph. Quality evaluation of included randomised controlled trials.

**Fig 3:**
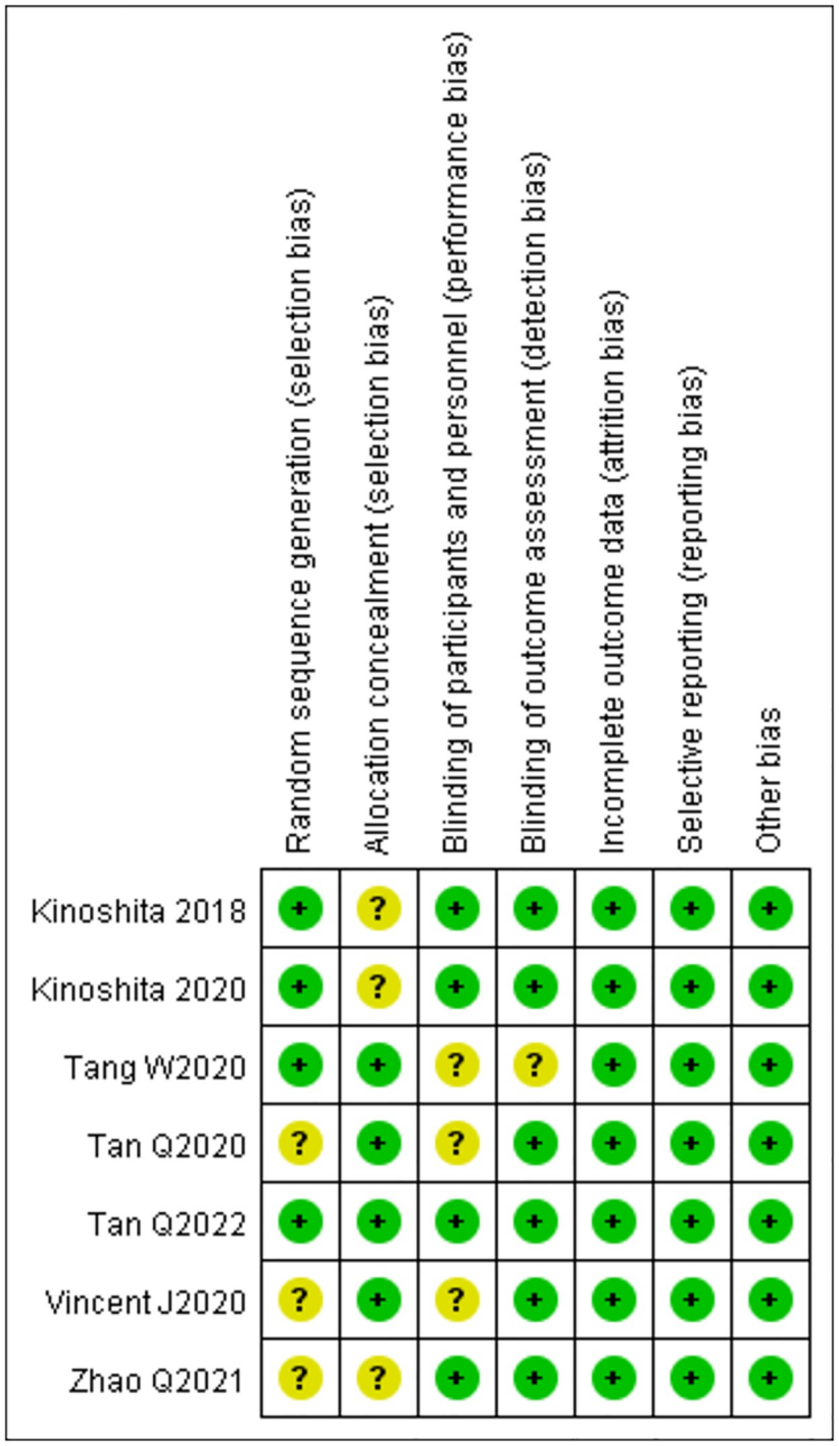
Risk of bias summary. Quality evaluation of included randomised controlled trials.

According to the Cochrane Collaboration’s tool for assessing the risk of bias, the quality of the RCTs was high (Figs 2 and 3). Four studies adopted randomization methods [17, 18, 20, 22], while the remaining studies did not. Only four studies used blinding methods [20–23]. The included studies completely reported all outcome data. Other biases in all the studies were regarded as low risk. The Newcastle-Ottawa Scale was used to evaluate the cohort studies. The three cohort studies scored six, six, and eight, respectively on the Newcastle-Ottawa Scale, and therefore, were assessed to be of moderate quality (Table 2). The MINORS was used to evaluate the non-randomized studies, and all studies demonstrated a high quality with a score of 19 (Table 3).

**Table 2:** Results of quality assessment using the Newcastle-Ottawa scale for cohort studies.

	Selection				Comparabiliy	Outcome			Score
	Representativeness of the exposed cohort	Seletion of the non exposed cohort	Ascertainment of exposure	Demonstration that outcome of interest was not present at start of study	Comparability of cohorts on the basis of the design or analysis	Assessment of outcome	Was follow-up long enough for outcomes to occur	Adequacy of follow up of cohorts	
Chen Z 2019	*	*	*	-	-	*	*	*	6
Chen Z 2021	*	*	*	-	*	*	*	*	8
Ji N 2022	*	*	*	-	*	*	*	*	8

A study can be awarded a maximum of one asterisk for each numbered item within the Selection and Outcome categories. A maximum of two asterisks can be given for Comparability.

**Table 3:** The revised and validated version of MINORS.

Methodological items for non-randomized studies	Score†		
	Luo Y2021	Niu YL2019	Jing J2018
1. **A clearly stated aim**: the question addressed should be precise and relevant in the light of available literature	2	2	2
2. **Inclusion of consecutive patients**: all patients potentially fit for inclusion (satisfying the criteria for inclusion) have been included in the study during the study period (no exclusion or details about the reasons for exclusion)	1	1	1
3. **Prospective collection of data**: data were collected according to a protocol established before the beginning of the study	2	2	2
4. **Endpoints appropriate to the aim of the study**: unambiguous explanation of the criteria used to evaluate the main outcome which should be in accordance with the question addressed by the study. Also, the endpoints should be assessed on an intention-to-treat basis.	2	2	2
5. **Unbiased assessment of the study endpoint**: blind evaluation of objective endpoints and double-blind evaluation of subjective endpoints. Otherwise the reasons for not blinding should be stated	0	0	0
6. **Follow-up period appropriate to the aim of the study**: the follow-up should be sufficiently long to allow the assessment of the main endpoint and possible adverse events	2	2	2
7. **Loss to follow up less than 5%**: all patients should be included in the follow up. Otherwise, the proportion lost to follow up should not exceed the proportion experiencing the major endpoint	2	2	2
8. **Prospective calculation of the study size**: information of the size of detectable difference of interest with a calculation of 95% confidence interval, according to the expected incidence of the outcome event, and information about the level for statistical significance and estimates of power when comparing the outcomes	0	0	0
*Additional criteria in the case of comparative study*			
9. **An adequate control group**: having a gold standard diagnostic test or therapeutic intervention recognized as the optimal intervention according to the available published data	2	2	2
10. **Contemporary groups**: control and studied group should be managed during the same time period (no historical comparison)	2	2	2
11. **Baseline equivalence of groups**: the groups should be similar regarding the criteria other than the studied endpoints. Absence of confounding factors that could bias the interpretation of the results	2	2	2
12. **Adequate statistical analyses**: whether the statistics were in accordance with the type of study with calculation of confidence intervals or relative risk	2	2	2
总分	19	19	19

^†^The items are scored 0 (not reported), 1 (reported but inadequate) or 2 (reported and adequate). The global ideal score being 16 for non-comparative studies and 24 for comparative studies.

### Atropine monotherapy group

#### Change in AL

All four studies analyzed the changes in AL with 0.01% atropine alone and OK alone. Based on an I2 of 16%, the fixed-effects model was selected for data merging. The WMD in the change in AL was -0.00 mm (95% CI: −0.05–0.04), which was statistically significant (p = 0.31). The meta-analysis indicated that the change in AL in the 0.01% atropine group was similar to that in the OK group.

**Fig 4:**
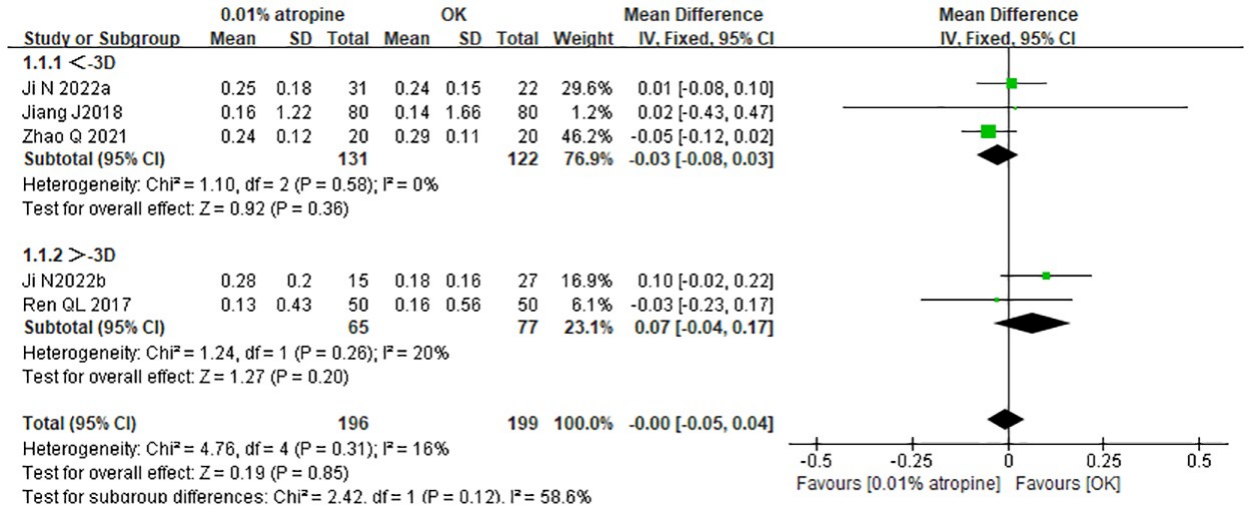
Forest plot of WMD of axial elongation in the atropine monotherapy group and the ortho-k monotherapy group and subgroup analysis by SER. WMD, weighted mean difference.

We performed a subgroup analysis according to whether the SER was greater than 3, to examine the effect of the baseline myopic degree on axial elongation. The corresponding WMDs for the aforementioned subgroups were as follows: −0.03 mm (95% CI: −0.08–−0.03) and 0.07 mm (95% CI: −0.04–0.17), respectively. These results indicated that the mean axial elongation in the 0.01% atropine alone group was similar to that in the OK alone group (Fig 4).

### Atropine combined with ortho-k therapy group

#### Change in AL

All 11 studies reported changes in the AL at baseline versus different follow-up periods in the AOK and OK groups. Of note, we chose a random-effects model because of the significant heterogeneity (I2 = 95%).

**Fig 5:**
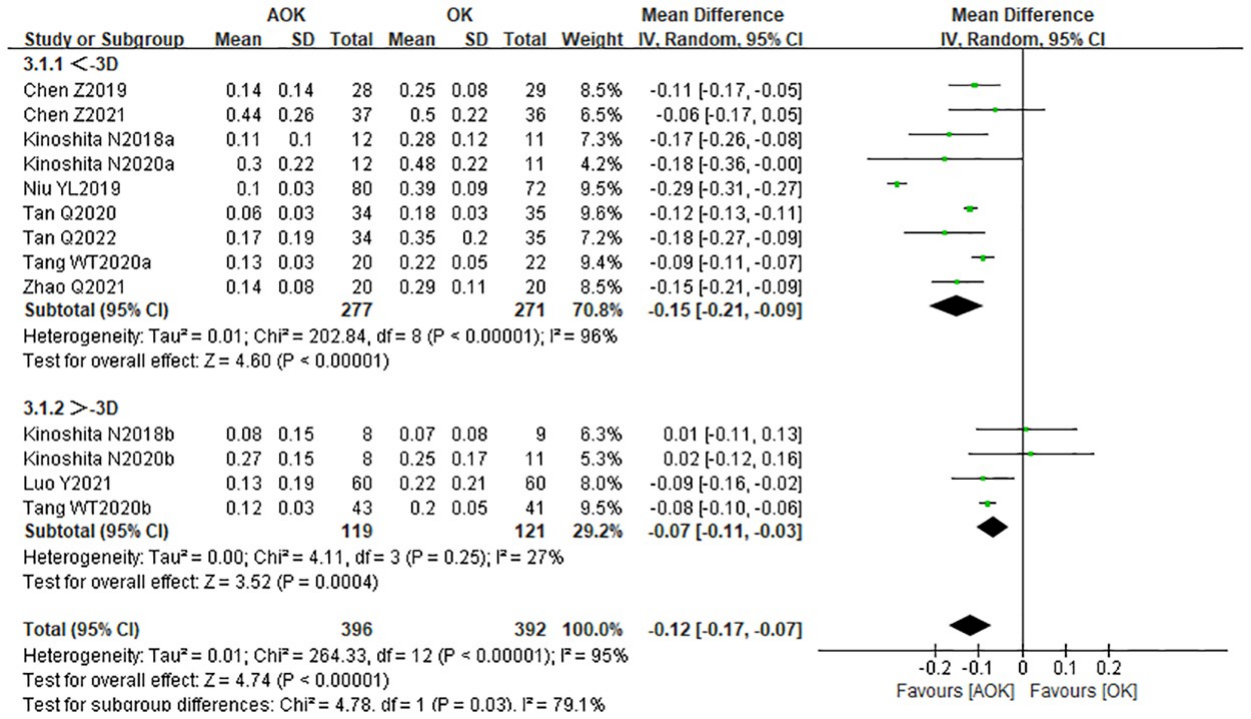
Forest plot of WMD of axial elongation in the AOK group and the OK group and subgroup analysis by baseline SER. WMD, weighted mean difference.

To investigate the effect of baseline refractive error (diopter) on axial elongation, we performed a subgroup analysis. We divided the included studies into two subgroups: low myopia (−1 to −3 D) and medium myopia (−3 to −6 D). There were nine studies with a baseline SER between −1 to −3 D. The combined results showed that the WMD in the low myopia subgroup was −0.15 mm (95% CI: −0.21–−0.09). There were four studies with a baseline SER between −3 to −6 D. The WMD of the medium myopia subgroup was −0.07 mm (95% CI: −0.11–0.03; Fig 5). The results confirmed that the AOK group significantly slowed axial elongation in children with low to moderate myopia, relative to the OK group.

**Fig 6:**
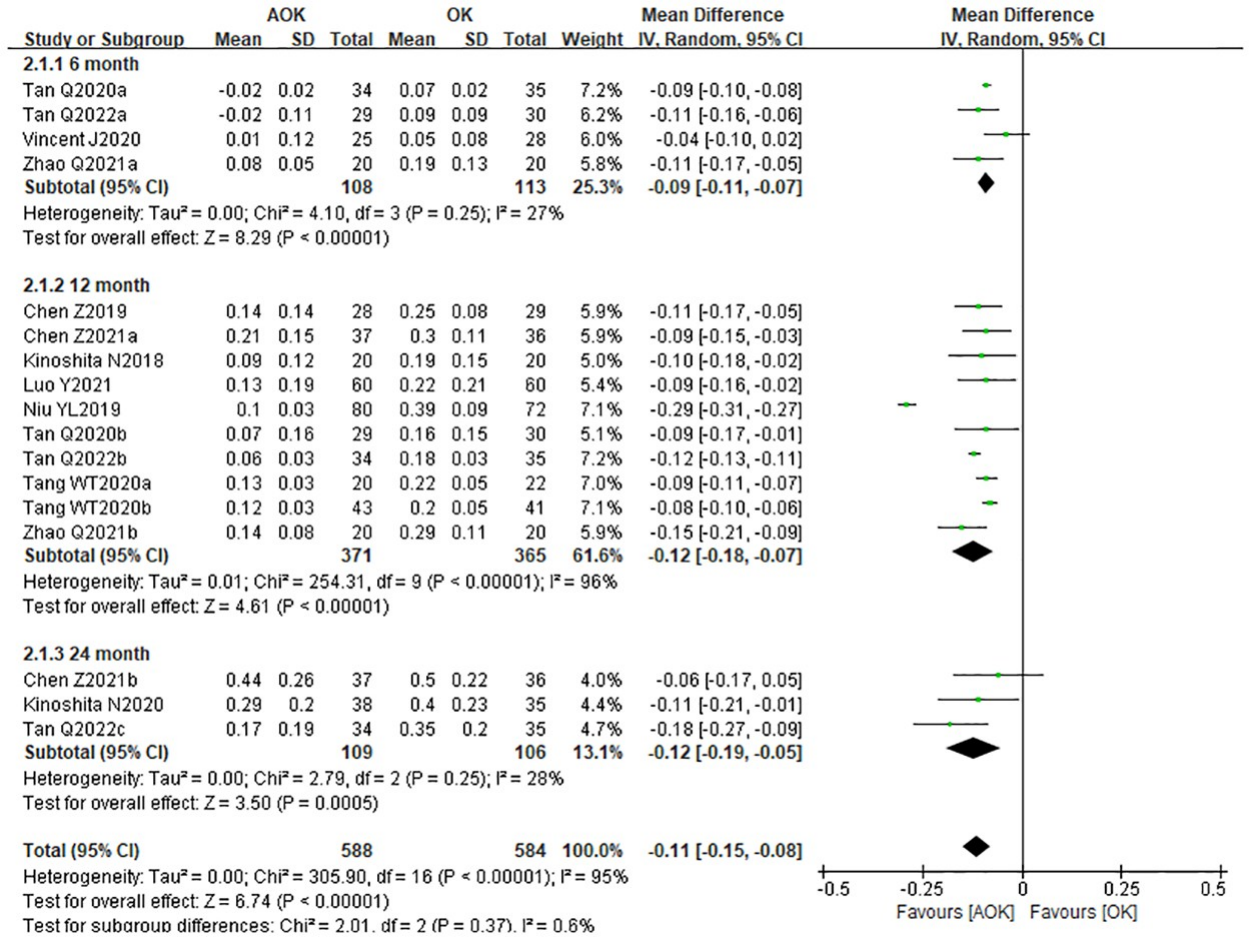
Forest plot of WMD of axial elongation in the AOK group and the OK group and subgroup analysis by follow-up periods. WMD, weighted mean difference.

We performed another subgroup analysis to investigate the impact of the follow-up period on axial elongation. The studies were divided into three groups according to the follow-up times of 6, 12, and 24 months. The corresponding WMD for each of the subgroups was as follows: −0.09 mm (95% CI: −0.11–−0.07), −0.12 mm (95% CI: −0.18–−0.07), and −0.12 mm (95% CI: −0.19–−0.05), respectively (Fig 6). The results indicated that AOK was more effective in slowing axial elongation than OK alone. The findings also suggest that the longer the follow-up period, the more significant and stable the effect of AOK on slowing the axial elongation.

#### Publication bias

**Fig 7:**
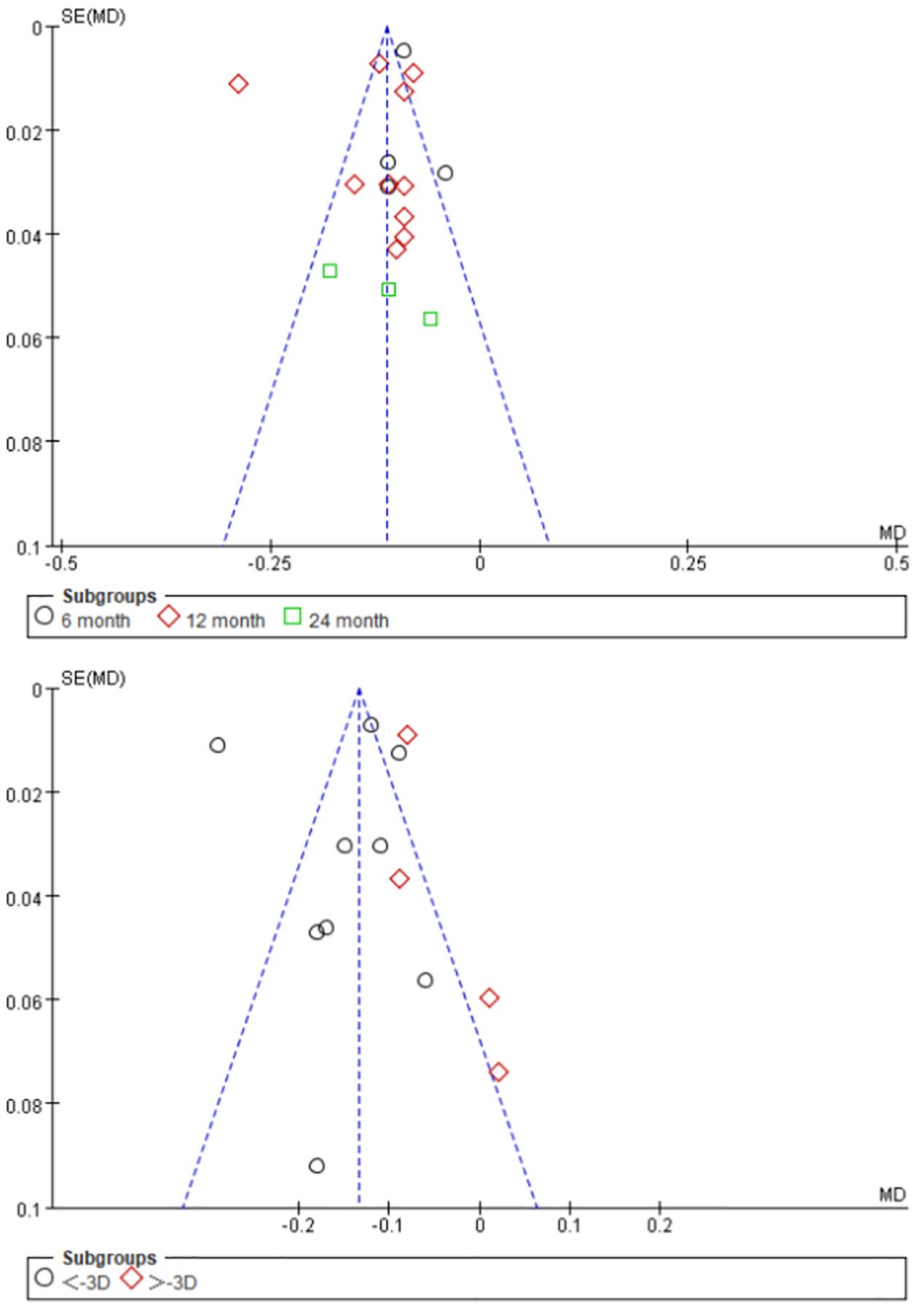
Funnel plot of axial length (AL).

A funnel plot was used to assess publication bias (Fig 7). The results showed no publication bias among the studies included in this meta-analysis.

#### Sensitivity analysis

A sensitivity analysis was conducted to estimate the effects of a single study on the overall results by removing one study at a time (Table 4). The analysis revealed that no single study significantly changed the overall results, thus, indicating that the pooled effect of the meta-analysis was stable and reliable.

**Table 4:** Subgroup sensitivity analysis.

	Total (95%CI)	Heterogeneity	Test for overall effect
Removal of Chen Z 2019	-0.13 [-0.14, -0.12]	I^2^ = 96%	P < 0.00001
Removal of Chen Z 2021	-0.13 [-0.14, -0.12]	I^2^ = 96%	P < 0.00001
Removal of Kinoshita N 2018	-0.13 [-0.14, -0.12]	I^2^ = 96%	P < 0.00001
Removal of Kinoshita N 2020	-0.13 [-0.14, -0.12]	I^2^ = 96%	P < 0.00001
Removal of Tan Q 2020	-0.14 [-0.15, -0.13]	I^2^ = 96%	P<0.00001
Removal of Tan Q2022	-0.13 [-0.14, -0.12]	I^2^ = 96%	P<0.00001
Removal of Tang WT2020	-0.14 [-0.15, -0.13]	I^2^ = 96%	P<0.00001
Removal of Zhao Q 2021	-0.13 [-0.14, -0.12]	I^2^ = 96%	P<0.00001
Removal of Niu YL2019	-0.10 [-0.11, -0.09]	I^2^ = 61%	P = 0.003
Removal of Luo Y2021	-0.13 [-0.14, -0.12]	I^2^ = 96%	P<0.00001
Removal of Vincent J2020	-0.11 [-0.12, -0.11]	I^2^ = 95%	P<0.00001

#### Change in SER

**Fig 8:**
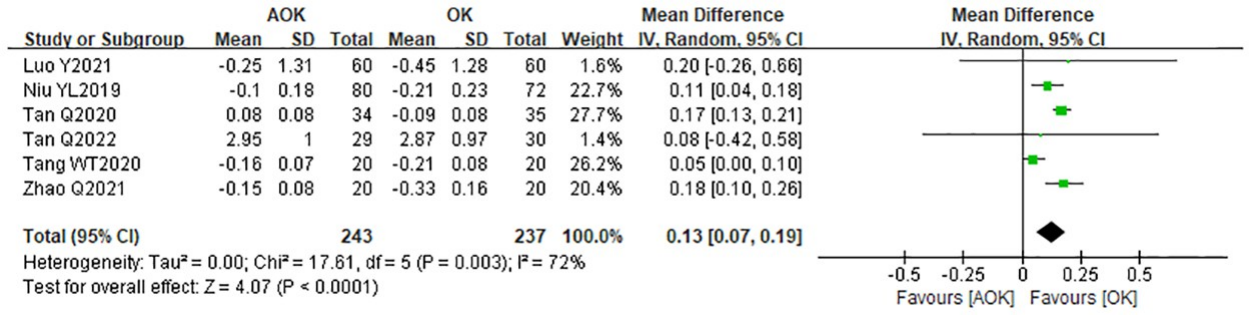
Forest plot of the comparison of change in SER. WMD, weighted mean difference.

Six studies compared OK with AOK in terms of the change in SER after treatment. Due to significant heterogeneity (I2 = 72%), a randomized effect model was selected, with the results showing WMD = 0.13 D (95% CI 0.07–0.19, p = 0.003; Fig 8). The results from this meta-analysis suggest that AOK is more effective in slowing SER progression than OK was.

#### Change in PD

**Fig 9:**
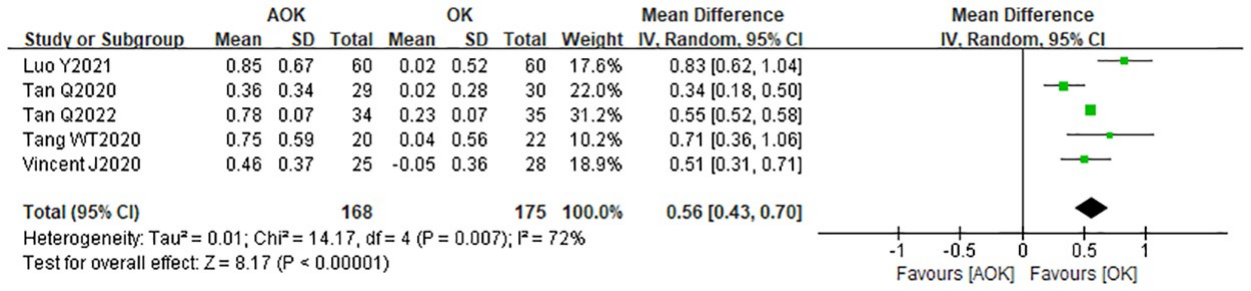
Forest plot of the comparison of change in PD. WMD, weighted mean difference.

Five studies compared OK with AOK in terms of the change in PD after treatment. Owing to significant heterogeneity (I2 = 72%), a randomized effect model was selected, with the results showing WMD = 0.56 mm (95% CI: 0.43–0.70, p = 0.007; Fig 9). The results suggest that AOK may slightly increase the PD, compared with OK alone.

#### Change in AA

**Fig 10:**
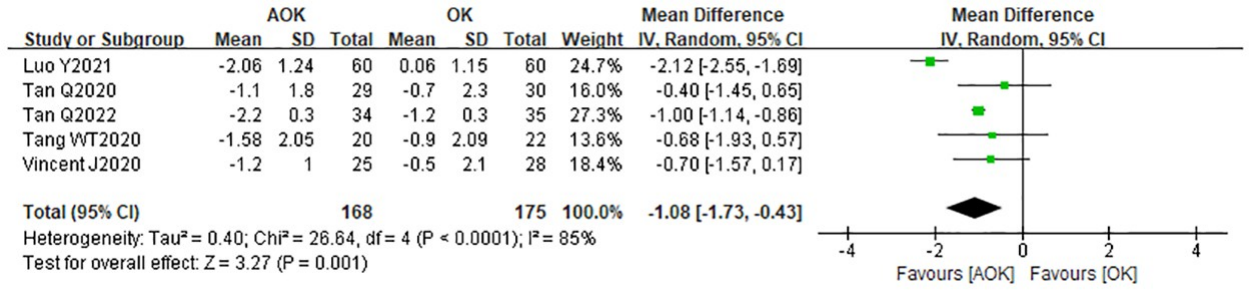
Forest plot of the comparison of change in AA. WMD, weighted mean difference.

Three studies included the change in AA. Based on an I2 of 85%, a randomized effects model was used. The WMD for the change in AL was −1.08 mm (95% CI: −1.73–−0.43), which was statistically significant (p<0.0001; Fig 10). These results suggest that AOK may slightly reduce the AA, compared with the effect of OK alone.

#### Change in IOP

**Fig 11:**
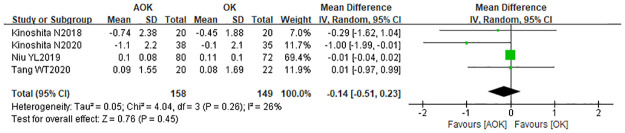
Forest the comparison of change in IOP. WMD, weighted mean difference.

The change in IOP between the two groups was reported by four studies. The combined results revealed a WMD of -0.14 (95% CI: −0.51–0.23), which suggested that the IOP was not significantly different between the two groups, as shown in Fig 11. Furthermore, there was no statistical heterogeneity between the two groups (p = 0.51, I2 = 0%).

#### Change in TBUT

**Fig 12:**
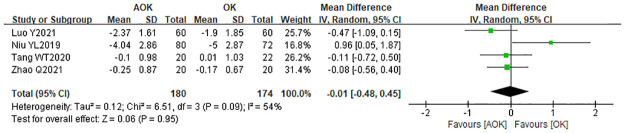
Forest plot of the comparison of change in TBUT. WMD, weighted mean difference.

Four studies reported the change in TBUT between the OK and AOK groups. These studies showed no statistical heterogeneity (p = 0.09). The results indicated WMD = −0.01 s (95% CI: −0.48–0.45), which suggests that TBUT was not significantly different between the AOK and OK groups (Fig 12).

#### Change in LLT

**Fig 13:**
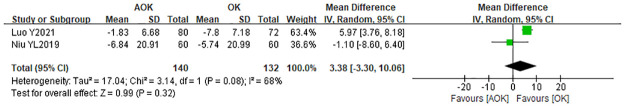
Forest plot of the comparison of change in LLT. WMD, weighted mean difference.

Two studies reported the change in LLT between the combination and monotherapy groups. The combined results indicated that there was no statistical difference in LLT between the two groups (95% CI: −3.30–10.06; Fig 13). Moreover, there was no significant between-study heterogeneity (p = 0.08, I2 = 68%).

#### Change in CECD

**Fig 14:**
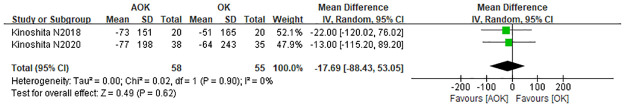
Forest plot of the comparison of change in CECD. WMD, weighted mean difference.

Two studies reported the change in CECD between the OK and AOK groups. The results showed WMD = –17.69 (95% CI: –88.43−53.05), which suggests that CECD did not differ significantly between the AOK and OK groups (Fig 14).

#### Change in UCVA

**Fig 15:**
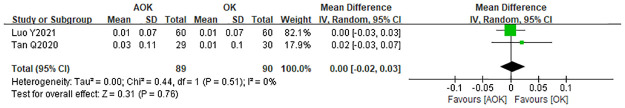
Forest plot of the comparison of change in UCVA. WMD, weighted mean difference.

Two studies included the change in UCVA. The results indicated no significant difference in UCVA between the groups (95% CI: –0.02−0.03; Fig 15). Also, no statistical heterogeneity was observed between the groups (p = 0.51, I2 = 0%).

#### Change in BCVA

**Fig 16:**
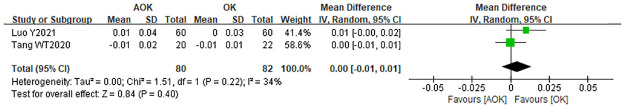
Forest plot of the comparison of change in BCVA. WMD, weighted mean difference.

Two studies reported the change in BCVA. The results indicated no significant difference in BCVA between the two groups (95% CI: –0.01−0.01), as shown in Fig 16. There was no statistical heterogeneity between the two groups (p = 0.90, I2 = 0%).

## Discussion

The results of this meta-analysis reveal that the axial growth rate of children who wear overnight OK alone is similar to that of children who wear single-vision spectacle lenses during the day and use one drop of 0.01% atropine before bedtime every night. Moreover, 0.01% atropine combined with OK is more effective than OK alone in slowing the axial elongation. The subgroup analysis demonstrated that the change in AL was influenced by the baseline myopic degree and duration of treatment. In contrast, there was no significant difference in the IOP, TBUT, LLT, CECD, UCDA, or BCDA between the AOK and OK treatment groups. However, the combination therapy is associated with a slight increase in PD and a decrease in the AA relative to OK therapy alone.

Presently, both atropine and OK are considered effective treatment strategies for myopia control. However, some controversies remain. Huang et al. reported that OK was worse than high- and medium-dose atropine, and equal to low-dose atropine for myopia control [29]. The latest SUCRA analysis revealed that in terms of hierarchy, OK was similar to 0.01%−0.025% atropine in AL inhibition over a one-year follow-up period [30]. There have been many reports that OK is more effective in slowing axial elongation in children with high myopia than in children with low myopia [31, 32]. Our meta-analysis shows that 0.01% atropine alone and OK alone have similar effects on slowing the axial elongation. However, with OK alone, the effect was greater in children with high myopia.

Synergistic effects of OK and atropine have been observed in previous studies [15–25]. Wan et al. demonstrated that the potential mechanisms underlying the combined effect of atropine and OK lenses relate to a large PD that increases retinal illumination, which in turn, lowers the myopic shift in the peripheral retina and enhances the effect of the OK lens [33]. Since all study participants wore OK lenses, we can assume that the difference between the two therapies, in terms of the change in AL, was primarily due to atropine. Low-concentration atropine can also lead to slight pupil enlargement, which may facilitate the optical effect of OK lenses [34]. Kinoshita et al. [17, 18] found that the additive effects of OK and 0.01% atropine on slowing axial elongation were greater in children with low myopia, whereas OK monotherapy was as effective as the combination therapy in children with high myopia [17, 18]. Conversely, the results of this meta-analysis conflicted with these previous studies. In this study, the effect of AOK in slowing axial elongation was greater in children with low myopia. This may be because the more peripheral myopia defocus was induced in high myopia compared with low myopia during OK treatment as described by previous studies. The effect of OK monotherapy in slowing the progression of high myopia may be adequate, whereas in children with low initial myopia, the amount of peripheral myopia defocusing may be insufficient. Additionally, the use of low-concentration atropine can compensate for the control effect of ortho-k in low myopia and contribute synergistically [17, 18, 31, 32].

AOK therapy significantly slowed the axial elongation at 6 and 12 months. However, when the treatment duration was extended to 24 months, the combination therapy did not lead to a further decrease in the axial elongation rate. Previous studies suggest that this may be due to temporary slight choroidal thickening after using atropine, which exaggerates the axial elongation control effect [35, 36]. Some studies also reported that the OK lens treatment effect was most marked within the first 12 months, and the effect becomes less marked but still significant in the second and third years [37].

No significant differences were found in the IOP, TBUT, LLT, CECD, UCDA, and BCDA between the AOK and OK groups, indicating that combination therapy for children with myopia has no negative influence on clinical results. The main complications of atropine use were photophobia, blurred vision, and allergic reactions [38]. The main complications of OK included infectious keratitis and allergic conjunctivitis [39]. These adverse reactions completely resolved when treatment stopped. Hence, the two interventions are considered relatively safe.

Our meta-analysis had some limitations. First, studies on 0.01% atropine alone and OK alone are still limited, and publication biases might have occurred. We only used axial elongation as the primary evaluation factor. We could not include refraction, PD, and other outcomes as the majority of the included studies did not report them. Second, due to the concentration dependence of atropine, different doses of atropine alone and OK alone must be analyzed. Third, the participants were all Asian children. The rate of myopia progression in Chinese children is generally higher than that in Caucasian children; thus, ethnicity may impact myopia progression and may influence the intervention effect. Future studies should consider ethnic differences. Finally, we did not consider all possible factors; for example, environmental and genetic factors, outdoor activities, and close work.

## Conclusions

For children with myopia, 0.01% atropine alone, and OK alone, have similar effects on slowing axial elongation. Conversely, 0.01% atropine combined with OK is more effective than OK alone in slowing axial elongation. Furthermore, baseline myopic degree and duration of treatment may affect the changes in axial elongation.

## Supporting information

S1 File PRISMA checklist. (DOC)

S2 File PRISMA flow diagram. (DOC)

S3 File Search strategy. (DOC)
