# Supplementary material for: The efficacy and safety of 0.01% atropine alone or combined with orthokeratology for children with myopia: A meta-analysis
Source: PLoS One. 2023 Jul 26;18(7):e0282286. doi: 10.1371/journal.pone.0282286 (PMC10370708; doi:10.1371/journal.pone.0282286)
Supplement: S2 File — (DOC) [file pone.0282286.s002.doc]

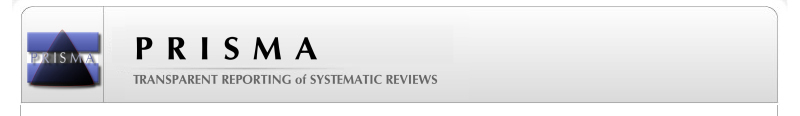
**PRISMA 2009 Flow Diagram**

**Screening**

**Included**

**Eligibility**

**Identification**

Records identified through database searching n = 549;PubMed= 367,EMABASE n=143,Cochrane library n=41

Additional records identified through other sources
(n = 0 )

Records after duplicates removed
(n =436 )

Records screened
(n = 121 )

Excluded(n=414):

1.Excluded via titles and abstracts(n=352)
2.Review，case report，meta-analysis(n=62)

Full-text articles assessed for eligibility
(n = 22 )

Excluded(n=8):

1.Treatment duration is less than 6 months (n=2)

2.Contain high myopia (n=3)

3.Atropine concentration > 0.1% (n=3)

Studies included in qualitative synthesis
(n = 14 )
