# Supplementary material for: The efficacy and safety of 0.01% atropine alone or combined with orthokeratology for children with myopia: A meta-analysis
Source: PLoS One. 2023 Jul 26;18(7):e0282286. doi: 10.1371/journal.pone.0282286 (PMC10370708; doi:10.1371/journal.pone.0282286)
Supplement: S3 File — (DOC) [file pone.0282286.s003.doc]

**Appendix .Search strategies modified in PubMed (a), Embase (b), and Cochrane CENTRAL (c)**

1. **Search strategy in PubMed**

| # | Search syntax | Citations found |
| --- | --- | --- |
| 1 | (myopia[MeSH Terms]) OR (near sight[Title/Abstract])OR (refractive errors[Title/Abstract])OR (nearsightedness[Title/Abstract]) |  |
| 2 | (orthokeratologic procedures[MeSH Terms])OR (orthokeratology[Title/Abstract])OR (ortho-k[Title/Abstract])OR (OK lens[Title/Abstract])OR (orthokeratology lens[Title/Abstract]） |  |
| 3 | (atropine[MeSH Terms])) OR (atropine sulfate[Title/Abstract]) |  |
| 4 | 1 AND 2 AND 3 | 367 |

1. **Search strategy in Embase**

| # | Search syntax | Citations found |
| --- | --- | --- |
| 1 | myopia/exp |  |
| 2 | near sight |  |
| 3 | refractive errors |  |
| 4 | nearsightedness |  |
| 5 | #1 OR #2 OR #3 OR #4 |  |
| 6 | orthokeratology/exp |  |
| 7 | orthokeratologic procedures |  |
| 8 | ortho-k |  |
| 9 | OK lens |  |
| 10 | orthokeratology lens |  |
| 11 | #6 OR #7 OR #8 OR #9 OR #10 |  |
| 12 | atropine/exp |  |
| 13 | atropine sulfate |  |
| 14 | #12 OR #13 |  |
| 15 | #5 and #11 and #14 | 143 |

1. **Search strategy in Cochrane library**

| # | Search syntax | Citations found |
| --- | --- | --- |
| 1 | MeSH myopia |  |
| 2 | near sight |  |
| 3 | refractive errors |  |
| 4 | nearsightedness |  |
| 5 | #1 OR #2 OR #3 OR #4 |  |
| 6 | MeSH orthokeratologic procedures |  |
| 7 | Orthokeratology |  |
| 8 | ortho-k |  |
| 9 | OK lens |  |
| 10 | orthokeratology lens |  |
| 11 | #6 OR #7 OR #8 OR #9 OR #10 |  |
| 12 | MeSH atropine |  |
| 13 | atropine sulfate |  |
| 14 | #12 OR #13 |  |
| 15 | #5 AND #11 AND #14 | 41 |
